# GFAP serves as a structural element of tunneling nanotubes between glioblastoma cells and could play a role in the intercellular transfer of mitochondria

**DOI:** 10.3389/fcell.2023.1221671

**Published:** 2023-10-11

**Authors:** L. Simone, D. L. Capobianco, F. Di Palma, E. Binda, F. G. Legnani, A. L. Vescovi, M. Svelto, F. Pisani

**Affiliations:** ^1^ Cancer Stem Cells Unit, Fondazione IRCCS Casa Sollievo della Sofferenza, San Giovanni Rotondo, Italy; ^2^ Department of Biosciences, Biotechnologies and Environment, University of Bari “Aldo Moro”, Bari, Italy; ^3^ Department of Neurosurgery, National Neurologic Institute IRCCS Besta, Milan, Italy; ^4^ Cellular Reprogramming Unit, Fondazione IRCCS Casa Sollievo della Sofferenza, San Giovanni Rotondo, Italy; ^5^ Institute of Biomembranes and Bioenergetics, National Research Council, Bari, Italy; ^6^ National Institute of Biostructures and Biosystems (INBB), Rome, Italy; ^7^ Center for Synaptic Neuroscience and Technology, Istituto Italiano di Tecnologia, Genoa, Italy

**Keywords:** cell-to-cell crosstalk, tunneling nanotubes, glioblastoma, GFAP, mitochondria

## Abstract

Tunneling nanotubes (TNTs) are long F-actin-positive plasma membrane bridges connecting distant cells, allowing the intercellular transfer of cellular cargoes, and are found to be involved in glioblastoma (GBM) intercellular crosstalk. Glial fibrillary acid protein (GFAP) is a key intermediate filament protein of glial cells involved in cytoskeleton remodeling and linked to GBM progression. Whether GFAP plays a role in TNT structure and function in GBM is unknown. Here, analyzing F-actin and GFAP localization by laser-scan confocal microscopy followed by 3D reconstruction (3D-LSCM) and mitochondria dynamic by live-cell time-lapse fluorescence microscopy, we show the presence of GFAP in TNTs containing functional mitochondria connecting distant human GBM cells. Taking advantage of super-resolution 3D-LSCM, we show the presence of GFAP-positive TNT-like structures in resected human GBM as well. Using H_2_O_2_ or the pro-apoptotic toxin staurosporine (STS), we show that GFAP-positive TNTs strongly increase during oxidative stress and apoptosis in the GBM cell line. Culturing GBM cells with STS-treated GBM cells, we show that STS triggers the formation of GFAP-positive TNTs between them. Finally, we provide evidence that mitochondria co-localize with GFAP at the tip of close-ended GFAP-positive TNTs and inside receiving STS-GBM cells. Summarizing, here we found that GFAP is a structural component of TNTs generated by GBM cells, that GFAP-positive TNTs are upregulated in response to oxidative stress and pro-apoptotic stress, and that GFAP interacts with mitochondria during the intercellular transfer. These findings contribute to elucidate the molecular structure of TNTs generated by GBM cells, highlighting the structural role of GFAP in TNTs and suggesting a functional role of this intermediate filament component in the intercellular mitochondria transfer between GBM cells in response to pro-apoptotic stimuli.

## Graphical Abstract

**Figure:**
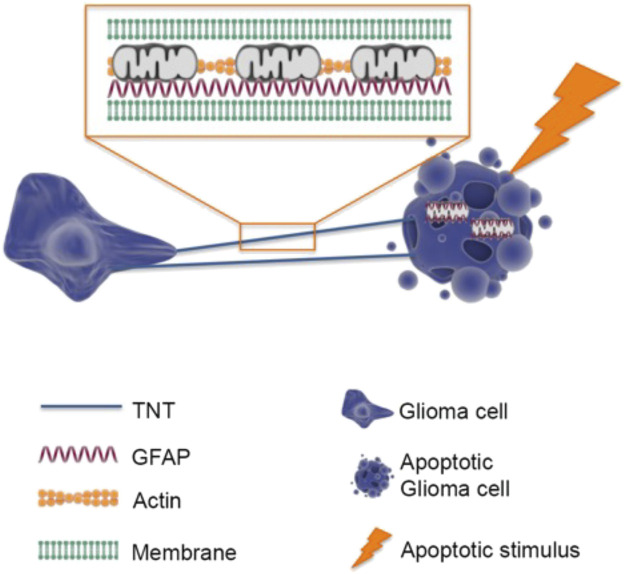
Intercellular trafficking of mitochondria among GBM cells is mediated by F-actin and GFAP-rich TNTs. In response to cell stress, GBM cells increase the number of TNTs where F-actin and GFAP represent the basic cytoskeletal elements. GFAP co-localizes with transferred mitochondria in receiving GBM cells.

## Introduction

It is well-known that a long-range crosstalk between cancer cells through paracrine signals and extracellular vesicles plays a central role in cancer progression (Broekman et al., 2018; Simone et al., 2022). Recently, a new form of long-range cell-to-cell crosstalk mechanism based on long plasma membrane bridges named tunneling nanotubes (TNTs) (Rustom et al., 2004) has emerged as a new mechanism involved in this context.

TNTs are described as non-adherent thin membranous protrusions ranging from 20 to 700 nm in width that directly connect two or more cells. They contain F-actin cytoskeletal filaments and are capable of transferring cargo between distant cells (Rustom et al., 2004; Cordero Cervantes and Zurzolo, 2021). TNTs allow for the intercellular transfer of not only small molecules such as ions, second messengers, and metabolic substrates but also of macromolecules including proteins, nucleic acids, and organelles. This results in a cell network that plays a role in the control of cancer progression (R et al., 2020). TNTs have been found in a variety of cancers, where they are utilized as cell mechanisms for the transfer of cellular material between cancer cells or within the tumoral microenvironment (Valdebenito et al., 2021).

In glioblastoma (GBM), the most prevalent and aggressive tumor in the central nervous system (CNS), TNTs have been recognized as a key feature of tumor progression (Pinto et al., 2020; Taiarol et al., 2021). TNT-mediated intercellular communication in GBM cell lines is affected by cell-stress stimuli, such as oxidative stress, cytotoxic treatments, and ionizing radiation, and they have been reported to contribute to tumoral progression and resistance due to the transfer of active signals (e.g., enzymes and mitochondria) from resistant to sensitive cells (Valdebenito et al., 2020).

Furthermore, TNTs are formed between GBM cells and tumor-associated stromal cells, contributing to the recruitment of healthy cells in the tumoral microenvironment. TNTs formed by GBM cells toward human primary astrocytes allow the transfer of mitochondria, resulting in the adaptation of non-tumor astrocytes to tumor-like metabolism and hypoxia conditions (Valdebenito et al., 2020; Valdebenito et al., 2021). Recently, TNT-based intercellular transfer of mitochondria was found to be correlated with GBM relapse, highlighting the potential role of TNT-mediated mitochondria transfer in GBM progression in different glioma stem-like cancer cells (Pinto et al., 2021). Consequently, understanding the molecular composition of TNTs and the mechanisms involved in TNT formation and mitochondria transfer could strongly contribute to the design of new anti-cancer strategies.

TNT-dependent mitochondrial transfer is known to be mediated by actin filaments, which represent the basic cytoskeletal elements of TNTs (Rustom et al., 2004). However, F-actin could be co-distributed with other representative cytoskeletal elements, such as microtubules (Jansens et al., 2017; Huang et al., 2022) or intermediate filaments (IFs), as demonstrated for cytokeratin 7 in cancer urothelial cells (Resnik et al., 2019).

The glial fibrillary acidic protein (GFAP) is a classic intermediate filament protein specific to astrocytes in the CNS (Guttenplan and Liddelow, 2019). It is widely used as a marker for reactive astrocytes, a condition in which astrocytes undergo morphological and functional remodeling in response to infections or pathological conditions (Liddelow and Barres, 2017; Escartin et al., 2021). GFAP is characteristic of astrocyte- and neural stem cell-derived gliomas in CNS tumors and is used to identify malignancies of glial origin, such as astrocytomas and GBM (Jung et al., 2007; Tichy et al., 2016; van Bodegraven et al., 2019a). Modifications in the structure and assembly of the GFAP network have been linked to a more malignant glioma phenotype (van Bodegraven et al., 2019b; Uceda-Castro et al., 2022). It is currently unknown whether GFAP plays a role in the structure and functions of TNTs in GBM cells.

In this study, we investigate the structural composition of TNTs and TNT-mediated mitochondria transfer between GBM cells in response to apoptotic stimuli. Our findings reveal that GBM cells generate F-actin and GFAP-positive and mitochondria-containing TNTs. We also observe GFAP-positive TNT-like structures within human GBM sections. We show that GFAP-positive TNTs are strongly upregulated by apoptotic stimuli; GFAP localizes at the tip of close-ended GFAP-positive TNTs and co-localizes with transferred mitochondria from GBM cells to receiving GBM-stressed cells.

Overall, our data demonstrate, for the first time, that GFAP not only serves as a marker of GBM progression but also plays a structural and functional role in TNT-mediated crosstalk between GBM cells.

## Results

### GBM cells are interconnected through GFAP-positive tunneling nanotubes containing active mitochondria

**FIGURE 1:**
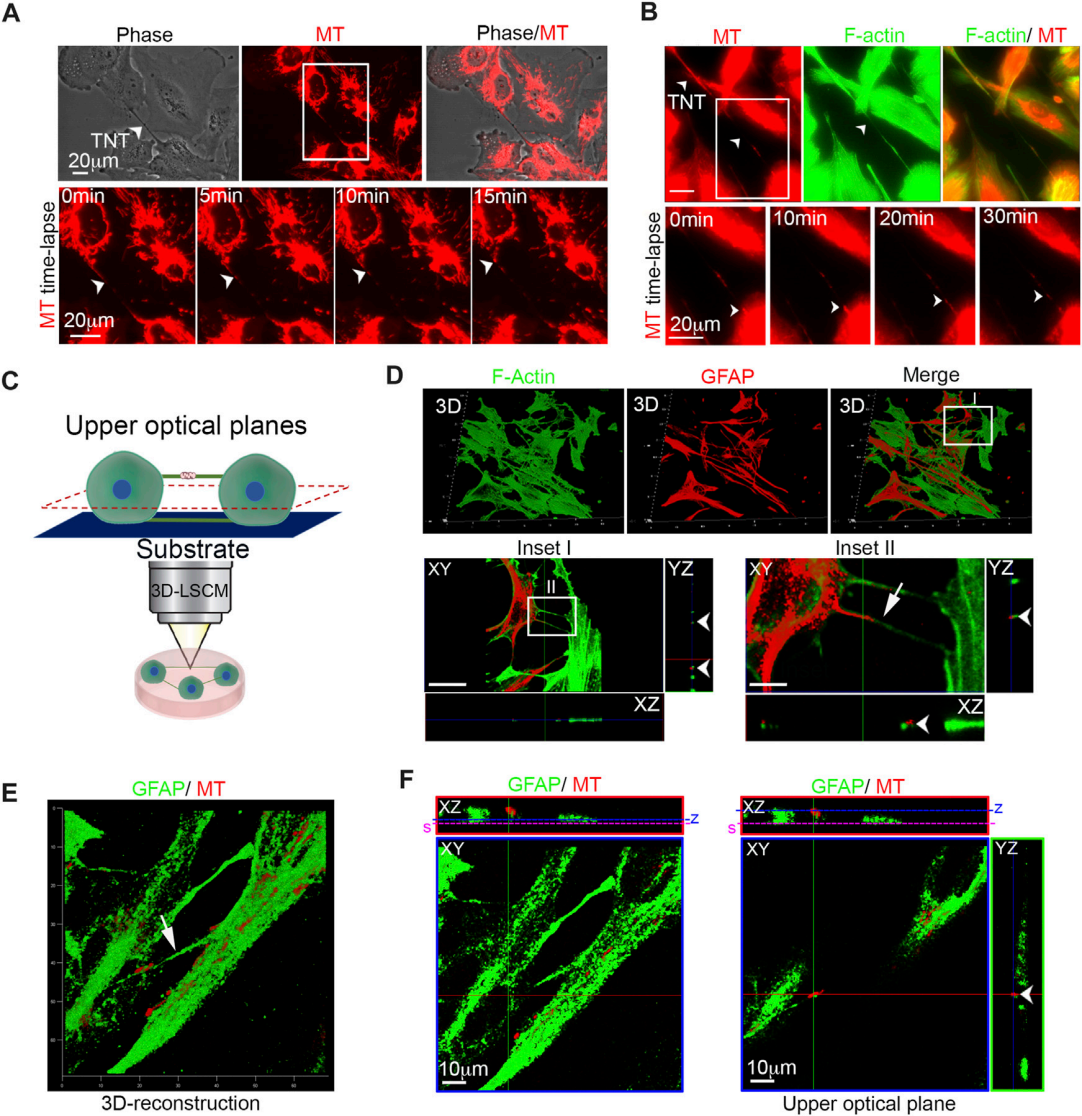
Glioma cells are connected to each other with GFAP-positive TNTs containing active mitochondria. **(A)** Live-cell imaging of mitochondria trafficking through TNTs. Mitochondria were stained with MitoTracker Red CMXRos (MT; red), and cells were analyzed by time-lapse fluorescence microscopy. Long intercellular bridges containing mitochondria were visible. The arrowheads indicate TNT. Scale bar: 20 μm. Bottom: The boxed area was enlarged. A series of time-lapse images taken at 5-min intervals is shown monitoring trafficking of mitochondria inside TNT. The arrowheads indicate the mitochondria running inside TNT. Scale bar: 20 μm ( Supplementary Movie S1). **(B)** Top: Live-cell time-lapse fluorescence microscopy of mitochondria trafficking through F-actin-positive TNTs. Mitochondria were stained with MitoTracker Red CMXRos (MT; red), and F-actin was stained with CellMask Green Actin Tracking Stain (green). The cells were analyzed by time-lapse fluorescence microscopy. The arrowheads indicate mitochondria that run inside F-actin-positive TNT. Scale bar: 20 μm. Bottom: The boxed area was enlarged. A series of time-lapse images taken at 10-min intervals is shown monitoring trafficking of mitochondria inside F-actin-positive TNT. The arrowheads indicate the mitochondria run inside the F-actin-positive TNT. Scale bar: 20 μm. (Supplementary Movie S2). **(C)** Pictorial representation describing laser-scan confocal microscopy, followed by 3D reconstruction and optical sectioning analysis (3D-LSCM). Only upper optical planes, detached from the substrate, were analyzed to correctly identify TNTs. **(D)** Top: 3D confocal reconstruction of TNTs connecting glioma cells stained with 488-conjugated phalloidin to visualize F-actin (green) and labeled with the anti-GFAP antibody (red) and relative Merge. Bottom: The boxed area (Inset I) is enlarged and XY plane and Z-projections displaying F-actin-rich TNTs. Scale bar: 25 μm. The boxed area in Inset I (Inset II) is enlarged and XY plane and Z-projections showing the presence of GFAP staining inside F-actin-rich TNTS. Scale bar: 5 μm. **(E)** Three-dimensional confocal reconstruction of glioma cells showing GFAP-positive TNTs (green) containing active mitochondria stained with ΔΨ-dependent MitoTracker Deep Red (MT; red). The arrow indicates GFAP-positive TNT. **(F)** Confocal single XY optical planes and Z-projections of 3D reconstruction reported in panel **(E)**. Two different XY optical planes are showed for two Z-positions (z, blue lines). Note that GFAP-positive TNT is detached from the substrate (s, pink line). Scale bar: 10 μm.

TNTs are defined as highly dynamic structures detached from the substrate, F-actin-positive, and able to transport cellular cargoes such as mitochondria. To investigate whether U87 glioma cells generate F-actin-positive TNTs that can transport cargoes, U87 cells were stained with MitoTracker Red CMXRos and CellMask Green Actin Tracking Stain. Time-lapse fluorescence microscopy of live cells was used to visualize the dynamics of mitochondria and the F-actin cytoskeleton. The images show that F-actin-positive TNTs rapidly transport mitochondria inside (Figures 1A, B; Supplementary Movies S1, S2).

To test whether F-actin-positive bridges were effectively detached from the substrate and to analyze GFAP localization, U87 cells were stained with phalloidin-488 to visualize F-actin, immunolabeled with the anti-GFAP antibody, and then were subjected to laser-scanning confocal microscopy followed by 3D reconstruction and optical sectioning analysis (3D-LSCM). Only the upper optical planes, which were detached from the substrate, were used for identifying TNTs (Figure 1C).

Our findings indicate that GBM cells are connected to each other through F-actin- and GFAP-positive TNTs (Figure 1D, bottom, insets I and II).

To determine whether GFAP-positive TNTs contain functional mitochondria, we stained U87 cells with fixable MitoTracker Deep Red and immunolabeled them for GFAP. 3D reconstruction shows that GFAP-rich TNTs contain functional mitochondria (Figure 1E). In detail, the lower (Figure 1F left) and the upper planes (Figure 1F right) of 3D reconstruction in Figure 1E show that TNT extends across the whole section and is detached from the substrate. The position of the two different XY optical planes is indicated in whole XZ projection.

In conclusion, our results demonstrate that glioma cells are interconnected through GFAP-positive TNTs containing active mitochondria.

### GFAP-positive TM-like and TNT-like structures are present in human GBM

**FIGURE 2:**
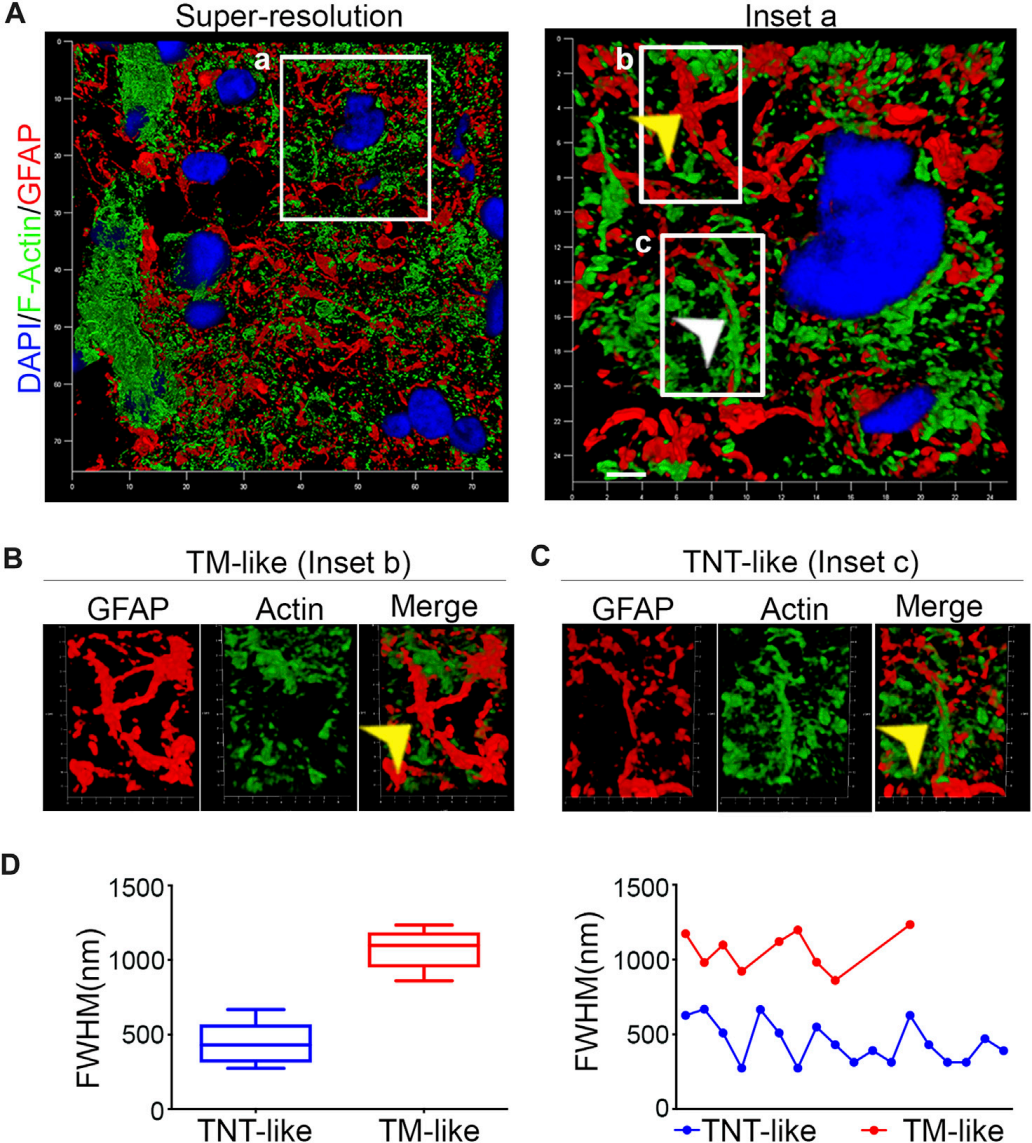
Human GBM contains GFAP-positive TM-like and TNT-like structures **(A)**. Confocal super-resolution images were obtained from resected human GBM tissues that were stained with phalloidin to visualize F-actin (green) and immunolabeled with the GFAP antibody (red). DAPI was used to stain the nuclei (blue). The boxed areas were enlarged to show different GFAP-positive structures **(B, C)**. The arrows indicate a GFAP-positive TM-like structure in Inset B and a GFAP-positive TNT-like structure in Inset **(C)**. Scale bar: 500 nm. **(D)**. Quantitative morphometric analysis showing the diameter of GFAP-positive structures found in GBM sections in **(A)**. The analysis of distribution of the diameters reveals the presence of two distinct populations of GFAP-positive structures: TNT-like structures with a medium diameter of <700 nm (blue) and TM-like structures with a medium diameter of >700 nm (red).

To investigate the presence of GFAP-positive TNTs in human brain tumors, resected WHO grade IV gliomas were stained for GFAP/F-actin and analyzed by super-resolution confocal microscopy. The obtained images were subjected to 3D reconstruction and quantitative morphometric analysis. We found numerous GFAP-positive “tube-like” signals within the GBM section (Figure 2A). Upon closer inspection using high-magnification 3D reconstruction images, we observed two distinct GFAP-positive structures resembling tumor microtube (TM)-like (R et al., 2020) and TNT-like structures (Figures 2B, C). Our morphometric analysis of the diameters of all GFAP signals revealed the existence of two separate populations: GFAP-positive TM-like protrusions with a medium diameter of >700 nm and GFAP-positive TNT-like structures with a medium diameter of <700 nm. The analysis of diameter size distribution shows that TMs and TNTs exhibit different patterns in size, with diameters ranging from 270 to 680 nm for TNTs and from 860 to 1240 nm for TMs (Figure 2D).

This super-resolution confocal microscopy analysis demonstrates the presence of both GFAP-positive TM-like structures and GFAP-positive TNT-like structures in human GBM sections.

### F-actin and GFAP-positive TNT formation between glioma cells increase during oxidative stress and apoptosis

**FIGURE 3:**
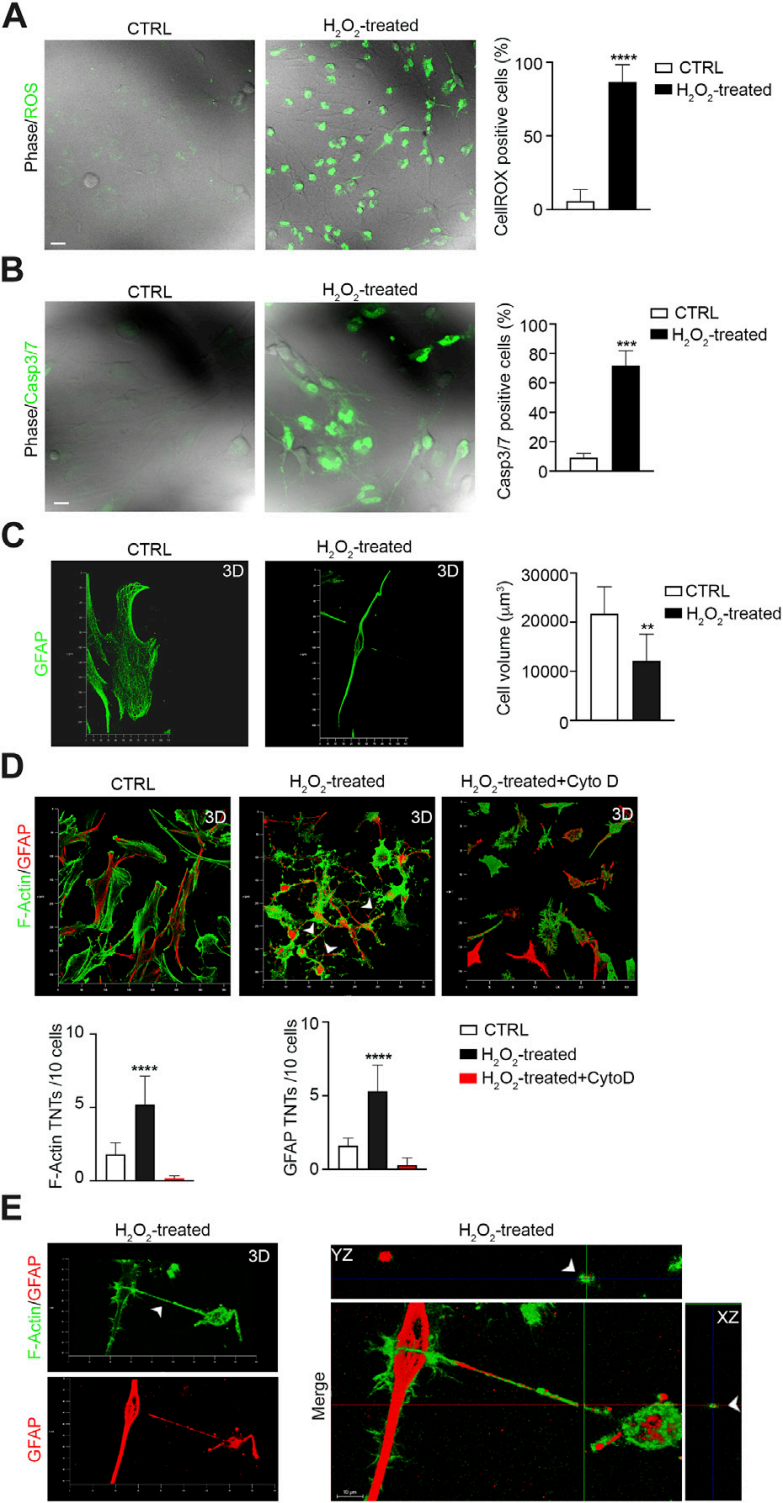
H_2_O_2_ treatment boosts F-actin- and GFAP-positive TNTs between GBM cells. **(A)** Representative confocal images of U87 untreated cells (CTRL) and treated with H_2_O_2_ (H_2_O_2_) and incubated with CellROX™ Green Reagent for ROS detection. Quantitative analysis of CellROX-positive cells for field. Values are expressed as mean ± SD of percentage of cells CellROX-positive on the total number of cells per field. ***p < 0.0001, *n* = 3. **(B)** Fluorescent images of caspase-3/7 activation in untreated cells (CTRL) and H_2_O_2_-treated U87 cells (H_2_O_2_-treated) stained with the CellEvent Casp3/7detection reagent (green). Scale bar: 10 μm. Quantitative analysis of Casp3/7-positive cells for field. Values are expressed as mean ± SD of the percentage of cells Casp3/7-positive on the total number of cells per field. ***p < 0,001, *n* = 3. **(C)** Confocal GFAP analysis and 3D reconstruction of the U87 cell volume of untreated cells (CTRL) and H_2_O_2_-treated cells (H_2_O_2_-treated). Histogram showing the values of the quantification of the cell volume of CTRL and STS-treated cells. Values are expressed as means ± S.E.M. **p < 0,005, n = 3. **(D)** The 3D confocal reconstruction shows U87 cells CTRL, and H_2_O_2_-treated or H_2_O_2_ + cytochalasin D-treated (H_2_O_2_-treated + Cyto D), as indicated in each lane. After treatment, the cells were stained with phalloidin to visualize F-actin (shown in green), immunolabeled with the GFAP antibody (shown in red) and analyzed by 3D-LSCM. Quantitative analysis of F-actin- and GFAP-positive TNTs in XY planes detached from the substrate. H_2_O_2_ triggers F-actin- and GFAP-positive TNTs, while cytochalasin D disrupts H_2_O_2_-induced TNTs. ****p < 0,0001, *n* = 3. **(E)** 3D confocal reconstruction of H_2_O_2_-treated cells. After treatments, the cells were stained with phalloidin to visualize F-actin (shown in green) and immunolabeled for GFAP (red). Merge and Z-projections of 3D reconstruction showing double positive TNTs.

Previous studies have shown that TNTs are induced by cell stress (Kretschmer et al., 2019; Raghavan et al., 2022). We investigated the effects of H_2_O_2_-induced oxidative stress on U87 cell–cell TNTs. We observed that H_2_O_2_ treatment strongly induced ROS production (Figure 3A) and apoptosis (Figure 3B), and resulted in a reduction in cell volume compared to untreated cells (Figure 3C). Analysis of the number of TNTs showed an increase in both F-actin- and GFAP-positive structures after H_2_O_2_ treatment. Cytochalasin D disrupts both F-actin- and GFAP-positive TNTs (Figures 3D, E). Moreover, the number of GFAP-positive cells also increased, following H_2_O_2_ treatment (Supplementary Figure S1).

**FIGURE 4:**
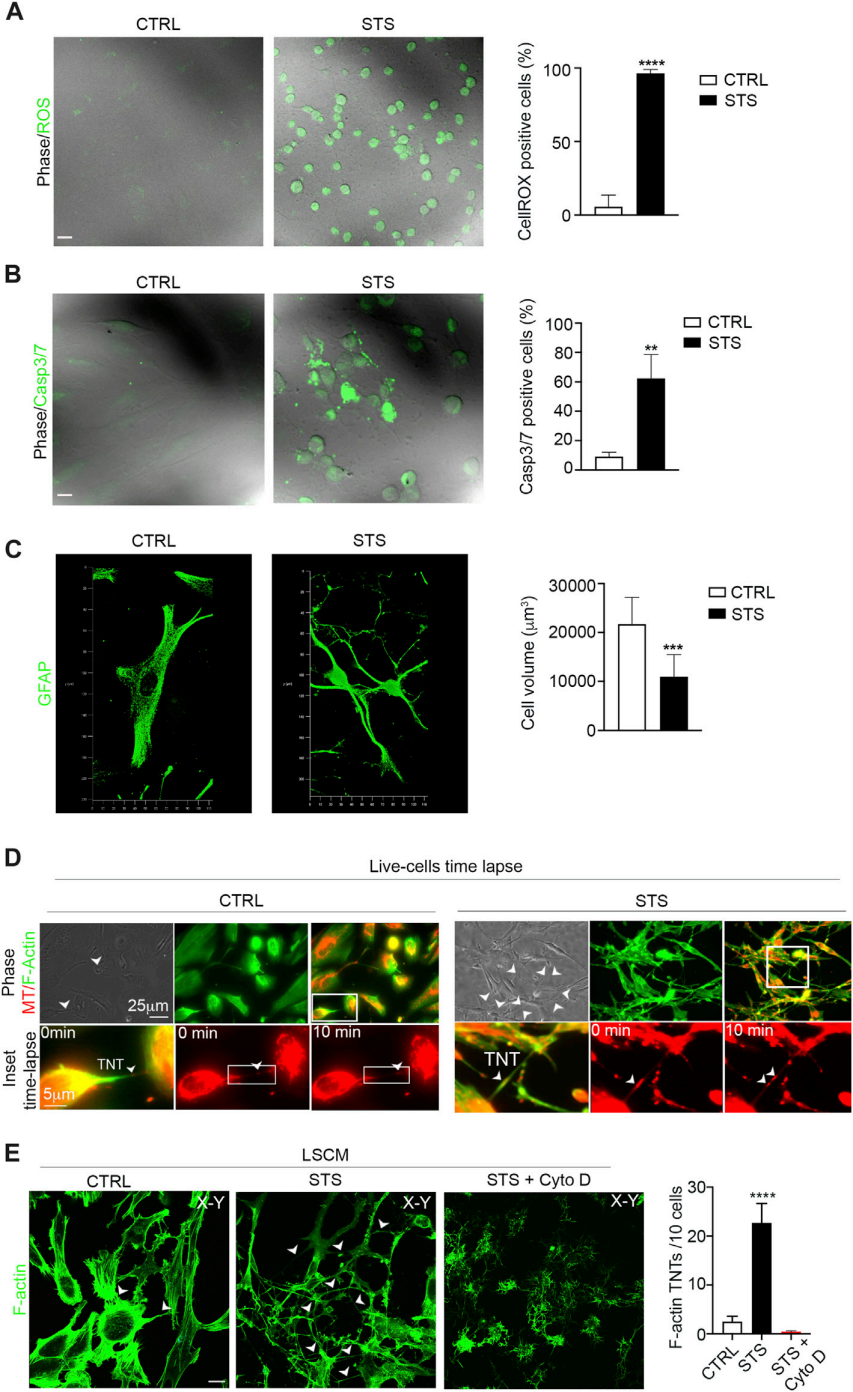
Staurosporine-induced apoptosis boosts F-actin-positive TNTs between GBM cells. **(A)** Representative confocal images of U87 untreated cells (CTRL) and treated with STS and incubated with CellROX™ Green Reagent for ROS detection. Quantitative analysis of CellROX-positive cells for field. Values are expressed as mean ± SD of the percentage of CellROX-positive cells on the total number of cells per field. ***p < 0.0001, *n* = 3. **(B)** Fluorescent images of caspase-3/7 activation in untreated cells (CTRL) and STS-treated cells (STS) stained with the CellEvent Casp3/7detection reagent (green). Scale bar: 10 μm. Quantitative analysis of Casp3/7-positive cells for field. Values are expressed as mean ± SD of the percentage of Casp3/7-positive cells on the total number of cells per field. **p < 0,005, n = 3. **(C)** Confocal GFAP analysis and 3D reconstruction of the cell volume of untreated cells (CTRL) and STS-treated cells (STS). Histogram showing the values of the quantification of the cell volume of CTRL and STS-treated cells. Values are expressed as means ± S.E.M. ***p < 0,001, *n* = 3. **(D)** Live-cell time-lapse fluorescence microscopy of mitochondria trafficking through TNTs in U87 culture under control conditions (CTRL) compared to staurosporine (STS)-treated U87 culture. Mitochondria were stained with MitoTracker Red (MT; shown in red), and F-actin was stained with CellMask Green Actin Tracking Stain (shown in green). The arrowheads show the TNT connections. Scale bar 25 μm. The boxed areas were enlarged to highlight the trafficking of mitochondria inside F-actin TNT structures (Supplementary Movies S3, S4). Scale bar: 5 μm. **(E)** Confocal images of CTRL, STS-treated, or STS + cytochalasin D treated U87 (STS + Cyto D), as indicated in each lane and stained with phalloidin showing the F-actin content. The cells were analyzed by 3D-LSCM. Representative XY planes, detached from the substrate, of CTRL and STS conditions. Note that STS treatment strongly upregulates F-actin-positive TNTs (arrowheads). Scale bar: 20 μm. Quantitative analysis of F-actin-positive TNTs in XY planes detached from the substrate. STS triggers F-actin-positive TNTs. ****p < 0,0001, *n* = 3.

To analyze whether apoptotic stress triggers GFAP-positive TNTs, we treated U87 cells with staurosporine (STS), a well-known pro-apoptotic cell stressor (Belmokhtar et al., 2001). After STS treatment, we first measured ROS production and Casp3/7 activation in STS-treated and untreated cells. The results showed that STS treatment strongly increased ROS levels (Figure 4A) and caspase3/7 activation (Figure 4B). The analysis of cell volume showed that STS-treated cells were significantly smaller in size than untreated counterparts (Figure 4C). Moreover, STS treatment led to an increase in the number of GFAP-positive cells (Supplementary Figure S1).

Based on these findings, we investigated whether glioma cells exposed to STS could form more canonical F-actin-positive TNTs containing functional mitochondria.

To visualize the dynamics of the F-actin cytoskeleton and mitochondria, we stained U87 cells with MitoTracker Red CMXRos and CellMask Green Actin Tracking Stain. Live cells were analyzed by time-lapse fluorescence microscopy, which revealed a significant upregulation of F-actin-positive TNTs that transport mitochondria in response to STS (Figure 4D, Supplementary Movies S3, S4). To quantify the number of TNTs, cells were fixed, stained with phalloidin to visualize F-actin, and analyzed by 3D-LSCM. The analysis of the number of F-actin TNTs detached from the substrate shows that STS treatment strongly induced the formation of F-actin-positive TNTs. Cytochalasin D treatment disrupts F-actin-positive TNTs (Figure 4E).

**FIGURE 5:**
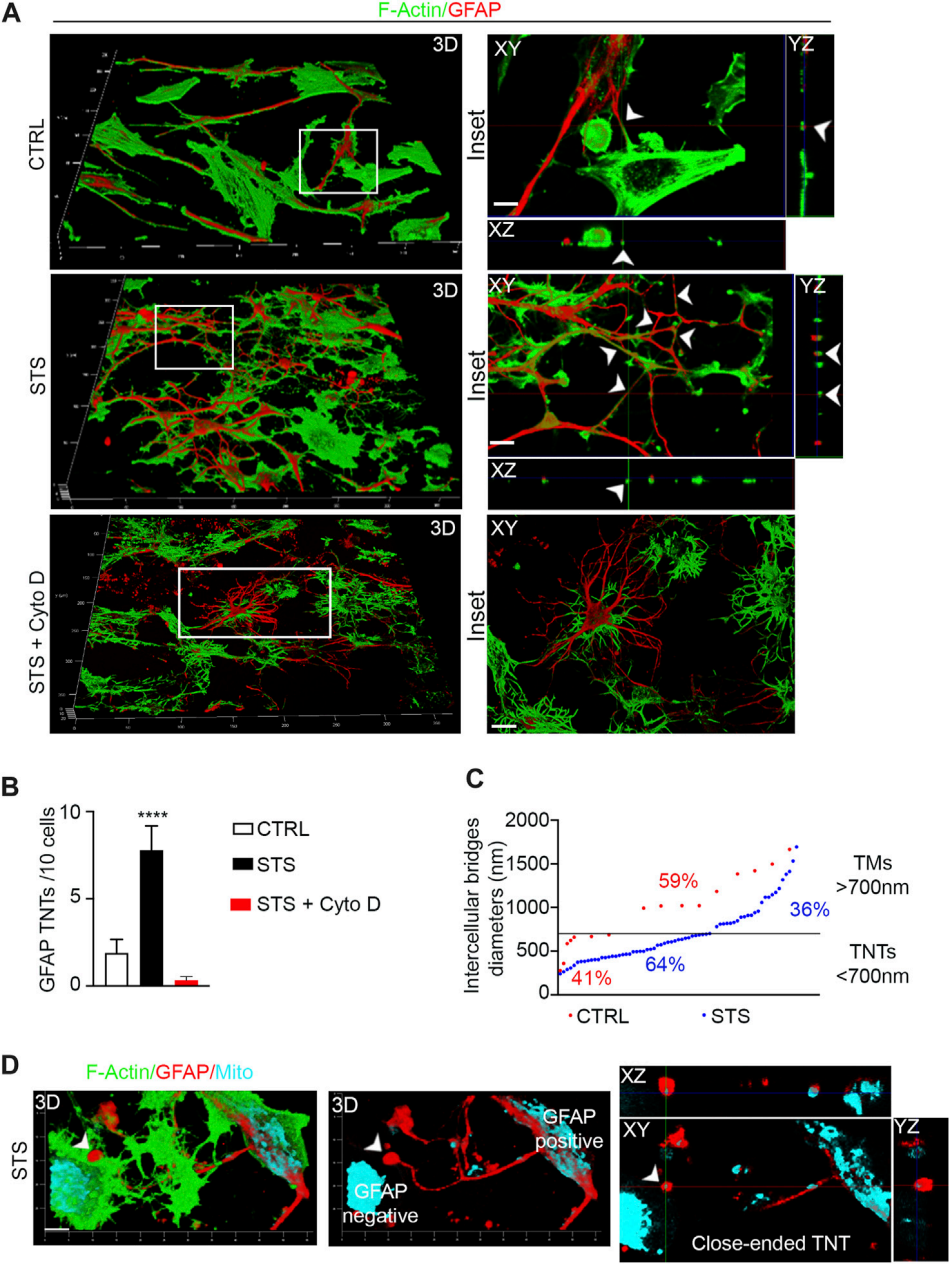
Staurosporine-induced apoptosis boosts GFAP-positive TNTs between GBM cells. **(A)** The 3D confocal reconstruction shows U87 cells CTRL, treated with staurosporine (STS) or STS + cytochalasin D (STS + Cyto D. After treatment, the cells were stained with phalloidin to visualize F-actin (shown in green) and GFAP antibody (shown in red), and analyzed by 3D-LSCM. The boxed areas were enlarged, and z-projections were generated to show TNTs rich in F-actin and GFAP. Scale bar: 10 μm. **(B)** Quantitative analysis of GFAP-positive TNTs in XY planes detached from the substrate. STS triggers GFAP-positive TNTs. ****p < 0.0001, *n* = 3. **(C)** Quantitative analysis of diameters of GFAP-positive intercellular bridges in CTRL (red) and STS (blue). In CTRL (red), the percentage of TNTs (<700 nm) was 41%, and the percentage of TMs (>700 nm) was 59%. In STS (blue), the percentage of TNTs (<700 nm) was 64%, and the percentage of TMs (>700 nm) was 36%. **(D)** Close-ended GFAP-positive TNT between U87 cells. The 3D confocal reconstruction shows STS-treated U87 cells stained for F-actin (green), GFAP (red), and mitochondria (cyano). The 3D reconstruction, single optical planes, and z-projections show a GFAP-positive intercellular bridge containing mitochondria, detached from the substrate and in contact with a GFAP-negative cell (left) through a spherical GFAP-positive and mitochondria-containing closed structure (arrowhead). This structure has the properties of a GFAP-positive close-ended TNT. Scale bar: 5 μm.

To investigate the contribution of GFAP-positive TNTs in response to pro-apoptotic stress, CTRL and STS-treated U87 cells were stained with phalloidin and immunolabeled for GFAP. The confocal analysis revealed a significant upregulation of GFAP-positive TNTs in response to STS. Cytochalasin D disrupts both F-actin- and GFAP-positive TNTs (Figure 5A). Additionally, GFAP-negative STS-treated cells also formed F-actin TNTs in response to STS (Supplementary Figure S2A).

The quantitative analysis of the number of GFAP-positive TNTs detached from the substrate shows that they are also significantly induced by STS and were completely destroyed by cytochalasin D treatment (Figure 5B). However, the F-actin/GFAP-positive TNT ratio is in favor of F-actin even after STS treatment. We further analyzed the diameters of GFAP-positive structures and found that the percentage of TNTs (<700 nm) was selectively upregulated in response to STS, while the percentage of TMs (>700 nm) was downregulated compared to the control condition (TNT 64% vs. TM 36% and TNT 41% vs. TM 59%, respectively) (Figure 5C).

When we analyzed the connection between glioma cells in more detail, we frequently observed GFAP-rich intercellular bridges containing mitochondria that were detached from the substrate and in contact with GFAP-negative cells with a spherical closed structure. This structure has the properties of a GFAP-positive close-ended TNT (Figure 5D).

These findings suggest that glioma cells respond to a pro-apoptotic signal by upregulating F-actin- and GFAP-positive TNTs.

### GFAP co-localizes with transferred mitochondria in response to pro-apoptotic stimuli

One key property of TNTs is their ability to mediate intercellular transfer of cargoes, such as organelles, between distant cells. Since we have observed that STS strongly upregulates GFAP-positive TNTs, we investigated whether GFAP plays an active role in the intercellular transfer of functional mitochondria from untreated GBM cells to GBM cells treated with STS.

**FIGURE 6:**
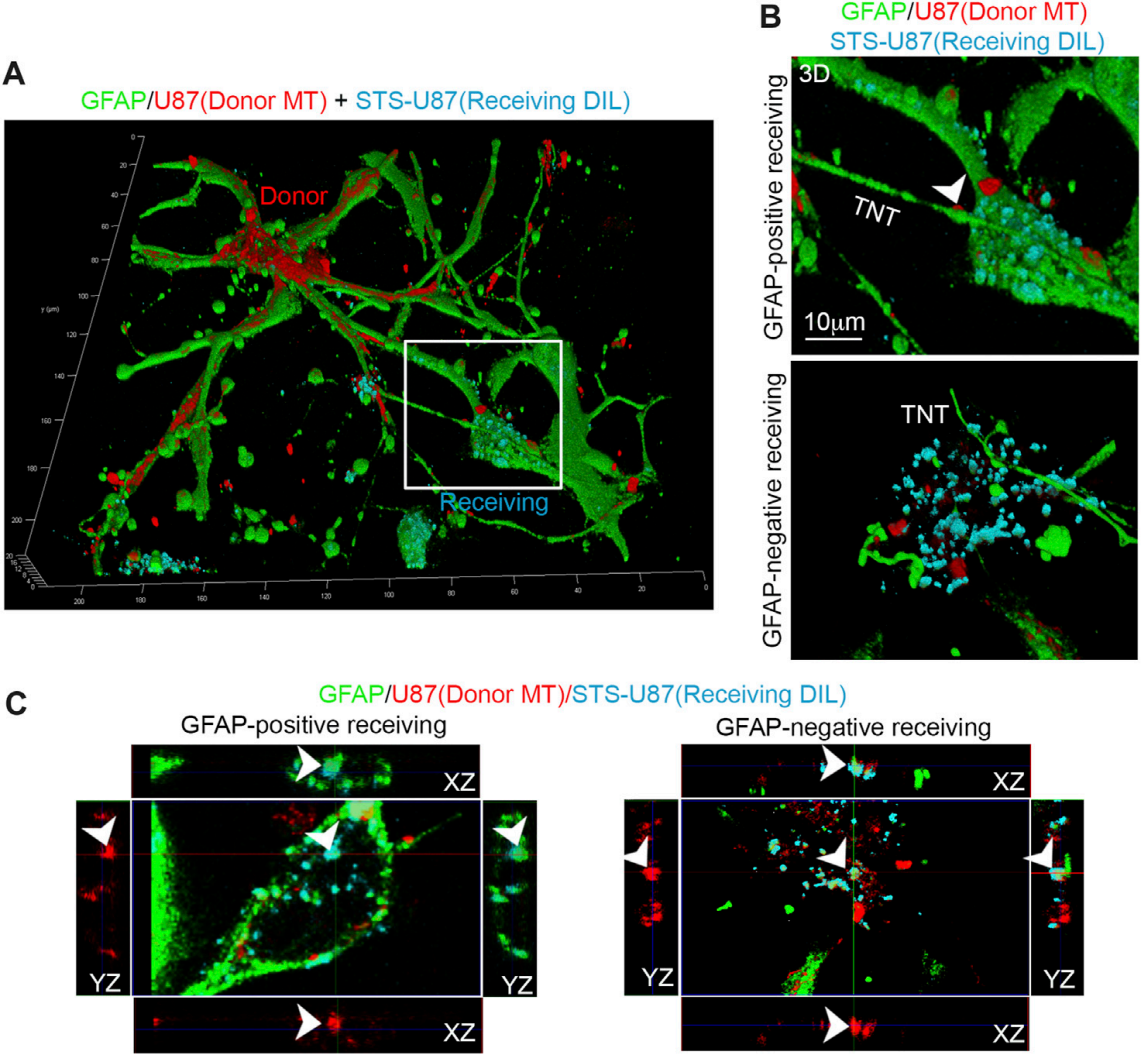
GFAP co-localizes with transferred mitochondria in receiving glioma cells. **(A)** U87 cells were stained with ΔΨ-dependent MitoTracker Deep Red to label mitochondria of untreated donor cells (donor MT, red). These cells were then co-cultured with U87 receiving cells that had been stained with a membrane tracker (receiving Dil, cyano) and treated with STS before the co-culture. After 24 h, the co-culture was fixed, immunolabeled for GFAP (green), and analyzed by 3D-LSCM. The inset indicates a GFAP-positive receiving cell. **(B)** Three-dimensional reconstruction of GFAP-positive and GFAP-negative receiving cells. Note the presence of GFAP-positive TNTs connected with both types of receiving cells and the presence of functional mitochondria inside TNT (arrowhead). Scale bar: 10 μm. **(C)** Confocal XY planes and Z-projections of receiving cells (receiving Dil, cyano) showing GFAP-positive (left) and GFAP-negative (right) receiving cells (Dil, cyano). Note the co-localization of GFAP (green), Dil (cyano), and MitoTracker Deep Red (red) signal (arrowheads) in both types of receiving cells.

To achieve this aim, we stained untreated U87 cells with a fixable and ΔΨ-dependent MitoTracker Deep Red (indicated as donor MT) and co-cultured them with STS-treated U87 cells that were previously stained with a plasma membrane dye Dil (indicated as receiving Dil). After 24 h, the co-culture was fixed, immunolabeled for GFAP, and analyzed through 3D-LSCM (Figure 6A). A deep analysis of 3D-LSCM images reveals the presence of GFAP-positive TNTs connected with both GFAP-positive and GFAP-negative U87 receiving Dil cells, in which functional mitochondria from untreated U87 cells are present (Figure 6B).

Moreover, GFAP-negative STS-treated cells are also interconnected with GFAP-positive untreated cells through F-actin-positive TNTs (Supplementary Figure S2B).

A more detailed XYZ slicing analysis of 3D-LSCM images shows that GFAP co-localizes with Dil and MitoTracker Deep Red in GFAP-positive and GFAP-negative receiving Dil cells (Figure 6C).

These data show that mitochondria and GFAP are co-transferred from untreated U87 cells to STS-treated U87 cells, suggesting a structural and functional role of GFAP in the intercellular transfer of mitochondria from untreated to suffering glioma cells.

## Discussion

GFAP has long been known to play a role in GBM, and mounting evidence highlights its involvement in GBM progression. For instance, GFAP expression levels are correlated with clinical outcomes and the cell fate regulation of GBM cancer stem cells (CSCs), as well as GBM-CSC invasion (Guichet et al., 2016; Ahmadipour et al., 2020; Sommerlath et al., 2022). GFAP overexpression in GBM is frequently associated with blood–brain barrier disruption, and it is commonly found in patients’ serum as a biomarker for gliomas (Yadav et al., 2022; Ghorbani et al., 2023). Alternative GFAP isoforms produced by alternative splicing have recently attracted attention for biomarker identification (Radu et al., 2022). It has been shown that GFAP splice variant isoform ratios modulate glioma cell invasion, cytoskeleton dynamic, and organization (Moeton et al., 2016; Stassen et al., 2017; van Bodegraven et al., 2019c; Uceda-Castro et al., 2022; van Asperen et al., 2022), suggesting that GFAP is not only a passive viewer in GBM but could also play an active role and highlight the correlation between GFAP and cytoskeleton remodeling in GBM cells.

The presence of TNTs between GBM cells and their correlation with GBM progression have already been investigated (Pinto et al., 2020), but whether GFAP plays a role in TNT-based crosstalk has not been explored yet, and the triggering signals of TNTs between GBM cells remain elusive. Civita and co-workers showed that TNT formation occurs in GFAP-positive reactive astrocytes when co-cultured with GBM cells. The authors investigated the formation of TNTs between astrocytes and GBM cells, analyzing GFAP as a marker of reactive astrocytes. However, whether and how GFAP plays a direct functional role in the formation of TNTs were not investigated (Civita et al., 2019). This is in line with a passive view of the GFAP’s role in TNT-mediated crosstalk.

In this study, we obtained data that could change this point of view, demonstrating that GFAP is a structural component of TNTs that connects GBM cells. Consistently with the data obtained between GBM cells in culture, we found GFAP-positive TNT-like and GFAP-positive TM-like structures in human GBM sections. Interestingly, in a mouse model of brain tumors, GFAP-positive intercellular astrocytoma membrane tubes with a width of over 1 µm and a length of more than 100 μm, called “tumor microtubes” (TMs), were found (Osswald et al., 2015). However, to the best of our knowledge, no data have been reported on GFAP-positive TMs and TNTs in human GBM. These data suggest the possibility that GFAP-positive TNTs could exist in human GBM.

We present the evidence that oxidative stress and pro-apoptotic stimuli upregulate GFAP-positive TNTs and induce the formation of close-ended GFAP-positive TNTs in GBM cells. We observed two different populations of GFAP-based intercellular connections based on their diameter: TM-like and TNT-like connections. Furthermore, we demonstrate that pro-apoptotic stimuli upregulate the ratio of GFAP-positive TNTs to TMs in GBM cells. Considering that TMs are always close-ended while TNTs are frequently open-ended, the upregulation of the TNT-dependent crosstalk could be useful to increase the intercellular transfer of large cargoes such as mitochondria contributing to stress response. Most interestingly, we found that GFAP co-localizes with mitochondria transferred from untreated GBM cells to stressed GBM cells.

These data highlight the structural role of GFAP in the formation of TNTs in response to pro-apoptotic stimuli in GBM and suggest a potential functional role of GFAP in the intercellular transfer of functional mitochondria in response to cell stress between GBM cells.

This new point of view is consistent with recent evidence about Alexander disease (AxD), a severe form of leukodystrophy mainly caused by GFAP mutation (Olabarria and Goldman, 2017). Intercellular communication mechanisms between astrocytes are compromised in AxD (Sofroniew, 2018). Gao and coworkers have shown that the presence of AxD-associated hotspot mutations (R79C and R239C) in the GFAP gene prevents intercellular mitochondria transfer between astrocytes (Gao et al., 2019). The authors discuss this evidence through different possibilities and cite TNTs as one potential mechanism involved, but whether GFAP mutation affects TNT formation was not investigated. Based on the data presented here, we can speculate that AxD-associated mutations could compromise the GFAP-dependent assembly of TNTs in astrocytes and, as a consequence, the intercellular transfer of mitochondria.

Our data also highlight the role of GFAP in the transfer of functional mitochondria from untreated GBM cells to apoptotic GBM-receiving cells. The activation of anti-apoptotic pathways in GBM is recognized as one of the main mechanisms regulating GBM progression. For instance, the Janus kinase/signal transducer and activator of the transcription (JAK/STAT) signaling pathway is known to control apoptosis and other pro-tumorigenic functions in GBM cells (Ou et al., 2021). Interestingly, mitochondria contribute to the regulation of this pathway (Kim et al., 2020). We can speculate that the GFAP-positive TNT-mediated transfer of functional mitochondria shown here could be involved in the well-known apoptotic escape mechanism that occurs in GBM cells.

Overall, our data reveal a new structural and functional role of GFAP in the crosstalk between GBM cells, demonstrating that GFAP participates in the formation of TNTs between GBM cells. Whether GFAP-positive TNTs occur between GBM cells and normal stromal cells and how this form of intercellular communication contributes to GBM progression will be further investigated. We believe that these new data shed new light on the role of this well-known protein in the study of GBM.

## Methods

### Cell lines

The cell line U87 MG (ATCC HTB-14, henceforth called U87), derived from a malignant glioma from a female patient by an explant technique, was acquired from ATCC. The cells were cultured in DMEM-F12 (1:1) (Gibco, cat # 11320033) supplemented with 10% FBS, 100 U/mL penicillin, and 100 mg/mL streptomycin, and maintained at 37°C in a 5% CO_2_ incubator.

### Antibodies

The following primary antibody was used: mouse anti-GFAP (Sigma-Aldrich, cat #IF03L) diluted to 1:500 dilution. The secondary antibodies used are as follows: donkey anti-mouse Alexa Fluor 488-conjugated (Thermo Fisher cat# A-21202) and donkey anti-mouse Alexa Fluor 647-conjugated (Thermo Fisher, cat #A-32787) diluted to 1:1000.

Furthermore, 488-conjugated phalloidin (Thermo Fisher, cat# A12379) and 647-labeled phalloidin (Thermo Fisher, cat# A22287) diluted to 1:500 were used to stain F-actin.

### Plasma membrane, actin, and mitochondria staining

To label the plasma membrane, the cells were incubated with 1:500 Vybrant Dil cell-labeling solution (Thermo Fisher, cat. #V22885) in a fresh medium for 30 min, then washed with serum-free DMEM-F12, and resuspended in a fresh medium.

To label mitochondria in living cells, the cells were incubated with 200 nM MitoTracker Red CMXRos (MT; Invitrogen, cat. #M7512) for 15 min; the cells were then washed with a prewarmed growth medium at least two times and left overnight in the growth medium.

To label mitochondria in fixed cells, the cells were incubated with 100 nM MitoTracker Deep Red FM (MT; Invitrogen, cat. #M22426) for 30 min; the cells were then washed with a fresh prewarmed growth medium at least two times and left overnight in the growth medium.

For live imaging of actin dynamics, the cells were incubated with CellMask Green Actin Tracking Stain (Thermo Fisher, A57243) diluted 1:1000 for 30 min in a serum-free medium; the cells were then washed with a low-serum medium.

### Cell culture treatment

U87 cells were seeded with a seeding density of 2 × 10^4^ cells in a 24 MW format and, the day after seeding, were incubated staurosporine (STS, cat. #S4400) at the final concentration of 2 μM for 2 h in a DMEM-F12 serum-free medium or with H_2_O_2_ 400 μM for 7h, then washed with a DMEM-F12 complete medium.

For co-culture experiments, the cells were subjected to plasma membrane staining before STS treatment.

### Live-cell imaging

For live-cell imaging, U87 cells were seeded in confocal dishes with a glass bottom (Greiner Bio-One, cat. #627870) and were subjected to the treatments or labeling, as described previously.

The phase contrast images, epifluorescence, and time-lapse were acquired using the BioStation IM-Q device, an incubator equipped with a microscope and a high-sensitivity cooled CCD camera. The acquisition conditions were as follows: 20× and 40× magnification 488- and 594-filter for excitation in the FluoroBrite DMEM (Thermo Fisher, A1896701).

To correctly visualize the mitochondria’ dynamics, images were acquired every 5–7 s with a low-intensity light source to reduce phototoxicity and photobleaching of the dye. Time-lapse images were analyzed using FIJI software.

## Immunofluorescence

To reduce the breakdown of TNTs and maintain the fluorescence of MitoTracker Deep Red, the cells were fixed directly in the medium by adding formaldehyde to a final concentration of 3.7% v/v for 15 min at 37°C. Afterward, the samples were carefully washed with PBS buffer, permeabilized using 0.3% Triton X-100 in PBS for 15 min, blocked with 3% BSA in PBS incubated for 1 h with the primary antibody, and washed with PBS/BSA buffer. The cells were finally incubated with Alexa Fluor–conjugated secondary antibodies. After careful washing, the samples were mounted in a VECTASHIELD antifade mounting medium (Vector Laboratories cat. #H-1000-10).

### GBM human samples

Fresh post-surgery high-grade glioma tissues were collected from patients with a confirmed diagnosis of GBM (WHO grade IV) enrolled at the National Neurologic Institute. “C. Besta” (Dr. F. Legnani). According to the ethical guidelines of the 2013 Declaration of Helsinki, they agreed to participate and signed an informed consent protocol accepted by the Ethical Committee of the National Neurologic Institute. “C. Besta” (protocol n 61). Primary human samples were postfixed in 4% paraformaldehyde for 24 h, and immunohistochemistry was performed on OCT-embedded 10-μm-thick serial sections and processed as previously described (Galli et al., 2004; Binda et al., 2017; Trivieri et al., 2022).

### Caspase-3/7 activity assay

U87 cells (4 × 10^4^ cells/well) were seeded in a 24-well plate and treated with STS 2 μM or H_2_O_2_ 400 μM or vehicles (DMSO or H_2_O) for 5 h. Then, the cells were incubated with 1 μL of the CellEvent™Caspase-3/7Green detection reagent (C10423, www.thermofisher.com) in 250 μL of serum-depleted medium for 30 min at 37°C in the dark for detection of the activity of caspase-3/7, according to the manual instructions. Stained cells were observed under an inverted fluorescence microscope. The values reported herein are the percentage of labeled cells/total cells per field of at least three independent experiments, and the error bars represent standard deviation of the mean.

### Epifluorescence and confocal microscopy

Fluorescence labeled cells were observed with a photomicroscope equipped for epifluorescence and 16× oil PL FL FLUOTAR objective, using the appropriate filter. Digital images were obtained with a DMX1200 camera (Nikon, Tokyo, Japan) and processed using LAS AF software (Leica Application Suite X). Once captured, the auto contrast function was applied to all the images using Photoshop CS5 (Adobe Photoshop).

Confocal microscopy was performed using a Leica TCS SP5 microscope. All acquisitions were performed by a serial acquisition mode between frames. XYZ-series were acquired with a raster size of 1024 × 1024 in the XY planes and a Z-step of 0.2 μm between optical slices. Three-dimensional (3D) images and projections from z-stack were constructed and processed using Leica Application Suite X software (LASX). Images were analyzed using LASX and FIJI software. The quantification of homotypic TNTs between U87 MG cells was performed as follows. Only F-actin/GFAP-positive structures, detached from support and connecting cells on single confocal planes, were counted. Randomly chosen images per biological replicate were acquired, and the TNT quantification was expressed as TNT number per field composed of 10/15 cells. Mitochondrial transfer in U87 MG/U87 MG cell co-cultures was analyzed on XYZ-series of confocal images using LASX software. U87 MG cell mitochondria, identified by MitoTracker Deep Red staining, were counted in XY intracellular U87 MG planes.

Confocal section’s images were captured using an STELLARIS 5 system. Post-processing was performed using LAS X LIGHTNING, and images were analyzed by LASX.

### GFAP-positive cell analysis

For GFAP-positive cell count, untreated or treated cells were mounted with a medium containing 50% glycerol, 1% n-propylgallate in PBS, and DAPI for nuclear staining.

Quantitative analysis was conducted on three to five different fields from each of three independent experiments. All the GFAP-positive cells in each field were properly counted and compared with nuclei number in each field using Fiji software.

### Analysis of reactive oxygen species generation

U87 cells (4 × 10^4^ cells/well) were seeded in a 24-well plate and treated with STS 2 μM or H_2_O_2_ 400 μM or vehicles (DMSO or H_2_O) for 5 h. Then, the cells were incubated with 5 μM CellROX Green Reagent (C10444, www.thermofisher.com) in a FBS-depleted medium for 30 min at 37°C in the dark, according to the manual instructions. Stained cells were observed under an inverted fluorescence microscope. The values reported herein are the percentage of labeled cells/total cells per field of at least three independent experiments, and the error bars represent standard deviation of the mean.

### Experimental design and statistical analysis

All data represent at least three replicates from independently prepared samples, as indicated in the figure legends. Statistical analyses were conducted using GraphPad Prism 6 software (GraphPad Prism). All data are reported as the mean ± SD. Statistically significant differences were computed using the Student’s *t*-test for unpaired data. The significance level was set at *p* < 0.05.

## Data Availability

The original contributions presented in the study are included in the article/Supplementary Material; further inquiries can be directed to the corresponding author.

## Abbreviations

3D-LSCM, 3D-laser scanner confocal microscopy; EMT, epithelial-to-mesenchymal transition; GBM, glioblastoma; GFAP, glial fibrillary acid protein; GSLCs, glioma stem-like cancer cells; TNTs, tunneling nanotubes; ROS, reactive oxidative species; STS, staurosporine.
