# Supplementary figures and images for: GFAP serves as a structural element of tunneling nanotubes between glioblastoma cells and could play a role in the intercellular transfer of mitochondria
Source: Front Cell Dev Biol. 2023 Oct 11;11:1221671. doi: 10.3389/fcell.2023.1221671 (PMC10598779; doi:10.3389/fcell.2023.1221671)

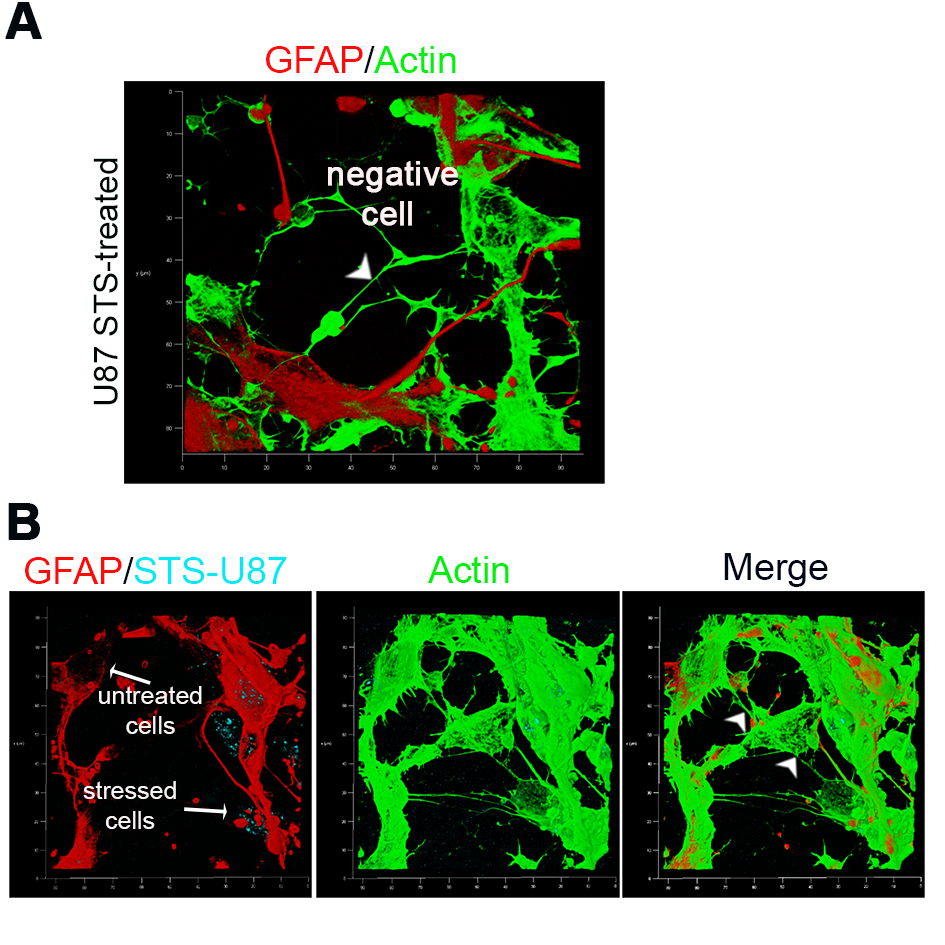

Supplement: Supplementary file 3 [file Image2.TIF]

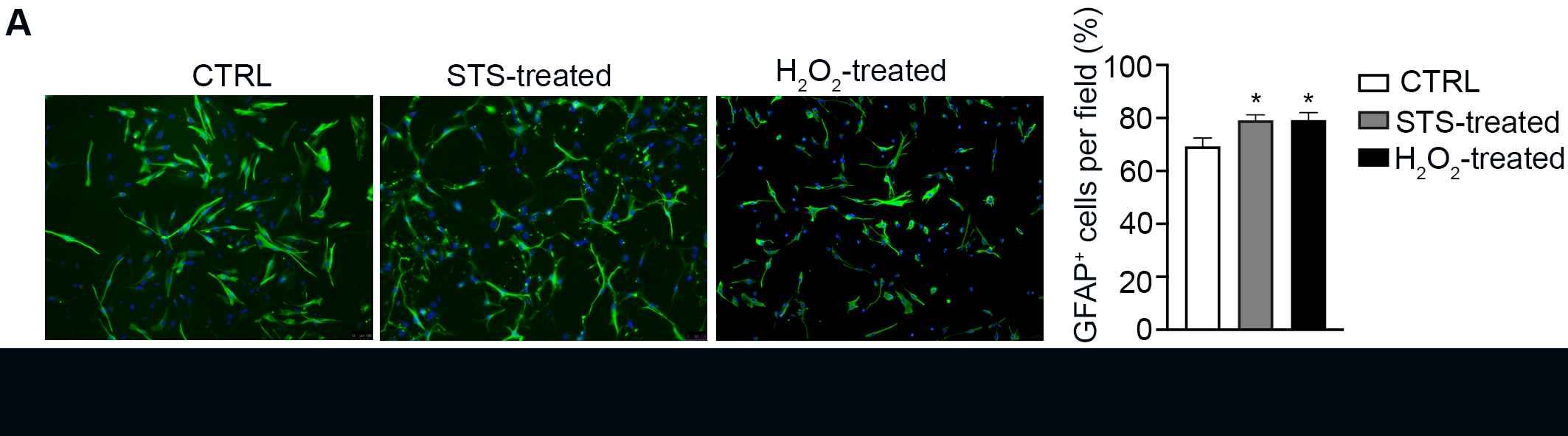

Supplement: Supplementary file 4 [file Image1.TIF]
