# Supplementary material for: GFAP serves as a structural element of tunneling nanotubes between glioblastoma cells and could play a role in the intercellular transfer of mitochondria
Source: Front Cell Dev Biol. 2023 Oct 11;11:1221671. doi: 10.3389/fcell.2023.1221671 (PMC10598779; doi:10.3389/fcell.2023.1221671)
Supplement: Supplementary file 6 [file DataSheet1.docx]

Supplementary Material

**GFAP serves as a structural element of tunneling nanotubes between glioblastoma multiforme cells and could play a role in the intercellular transfer of mitochondria**

**Simone L.^1^, Capobianco D.L.^2^, Binda E.^1^, Legnani F.^3^, Vescovi A.L.^4^, Svelto M. ^2,5^, Pisani F. ^2,6*^**

*** Correspondence:**

Pisani Francesco

francesco.pisani@uniba.it

**Supplementary Movie 1 (Figure 1A):** A series of time-lapse images taken at 5-min intervals is shown monitoring trafficking of mitochondria inside the TNT.

**Supplementary Movie 2 (Figure 1B):** A series of time-lapse images taken at 10-min intervals is shown monitoring trafficking of mitochondria inside the F-actin positive TNT

**Supplementary Movie 3 (Figure 3A):** The trafficking of mitochondria inside F-actin TNT in CTRL.

**Supplementary Movie 4 (Figure 3A):** The trafficking of mitochondria inside F-actin TNT in STS.

**Supplementary Figure 1: Effect of pro-apoptotic stimuli on GFAP expression**

Epifluorescence images of U87 untreated (CTRL) and treated with Staurosporine (STS) or H_2_O_2_. GFAP staining is shown in green and Dapi (blu). Quantitative analysis of GFAP positive cells for field. Values are expressed as mean ± SD of percentage of cells GFAP positive on the total number of cells per field. **p< 0,05*, n=3.

**Supplementary Figure 2: GFAP-negative cells STS-treated cells form F-actin positive TNT**

**A.** 3D confocal reconstruction shows STS-treated U87 cells. After treatment, cells were stained with Phalloidin to visualize F-actin (shown in green), immunolabeled with GFAP antibody (shown in red) and analyzed by 3D-LSCM. Note that GFAP-negative cell is interconnected with GFAP-positive cell

through F-actin positive TNTs.

**B.** 3D confocal reconstruction showing interconnection between STS-treated and untreated cells. Untreated cells were co-cultured with U87 receiving cells that had been stained with a membrane tracker (receiving DiI, cyano) and treated with STS before the coculture. After 24h the coculture was stained with Phalloidin (actin, green) and immunolabeled for GFAP (red) and analysed by 3D-LSCM. Note that the GFAP-negative STS-treated cell (stressed cells) is interconnected with GFAP positive untreated cells by long F-actin positive TNT.
